# The Comparative Toxic Impact Assessment of Carbon Nanotubes, Fullerene, Graphene, and Graphene Oxide on Marine Microalgae *Porphyridium purpureum*

**DOI:** 10.3390/toxics11060491

**Published:** 2023-05-30

**Authors:** Konstantin Pikula, Seyed Ali Johari, Ralph Santos-Oliveira, Kirill Golokhvast

**Affiliations:** 1 Polytechnical Institute, Far Eastern Federal University, 10 Ajax Bay, Russky Island, Vladivostok 690922, Russia; golokhvast@sfsca.ru; 2 Department of Fisheries, Faculty of Natural Resources, University of Kurdistan, Pasdaran St, Sanandaj 66177-15175, Iran; sajohari@gmail.com; 3 Laboratory of Nanoradiopharmaceuticals and Synthesis of Novel Radiopharmaceuticals, Nuclear Engineering Institute, Brazilian Nuclear Energy Commission, Rua Hélio de Almeida 75, Rio de Janeiro 21941906, Brazil; roliveira@ien.gov.br; 4 Laboratory of Nanoradiopharmaceuticals and Radiopharmacy, Rio de Janeiro State University, R. São Francisco Xavier, 524, Rio de Janeiro 23070200, Brazil; 5 Siberian Federal Scientific Center of Agrobiotechnology RAS, Centralnaya Str., Presidium, Krasnoobsk 633501, Russia

**Keywords:** carbon nanomaterials, graphene-family materials, bioassay, nanotoxicology, ecotoxicology, flow cytometry, growth rate inhibition

## Abstract

The growing production and application of carbon-based nanomaterials (CNMs) represent possible risks for aquatic systems. However, the variety of CNMs with different physical and chemical properties and different morphology complicate the understanding of their potential toxicity. This paper aims to evaluate and compare the toxic impact of the four most common CNMs, namely multiwalled carbon nanotubes (CNTs), fullerene (C60), graphene (Gr), and graphene oxide (GrO) on the marine microalgae *Porphyridium purpureum*. The microalgae cells were exposed to the CNMs for 96 h and measured by flow cytometry. Based on the obtained results, we determined no observed effect level (NOEL), and calculated EC10 and EC50 concentrations for growth rate inhibition, esterase activity, membrane potential, and reactive oxygen species (ROS) generation changes for each tested CNM. According to the sensitivity (growth rate inhibition) of *P. purpureum*, the used CNMs can be listed in the following order (EC50 in mg/L, 96 h): CNTs (2.08) > GrO (23.37) > Gr (94.88) > C60 (>131.0). The toxicity of CNTs was significantly higher than the toxic effect of the other used CNMs, and only this sample caused an increase in ROS generation in microalgae cells. This effect was apparently caused by the high affinity between particles and microalgae associated with the presence of exopolysaccharide coverage on *P. purpureum* cells.

## 1. Introduction

Production and research on carbon-based nanomaterials (CNMs) have undergone exponential growth over the last decade [1,2,3]. In 2021, the global CNM market was valued at USD 2.9 billion and projected to reach USD 31.6 billion by 2031, with a compound annual growth rate (CAGR) of 27.7%, according to a report by Allied Market Research (https://www.alliedmarketresearch.com/carbon-nano-materials-market (accessed on 5 May 2023)). The main representatives of the family of CNMs are fullerene (C60 or C70), graphene (Gr), graphene oxide (GrO), carbon nanotubes (CNTs), carbon quantum dots, and other derivatives [4]. Freixa et al. (2018) stated in their analytical work that fullerenes are the most studied group of CNMs, followed by multiwalled carbon nanotubes (MWCNTs), single-walled carbon nanotubes (SWCNTs), Gr, and black carbon [5]. The interest in CNMs is based on their unique mechanical, electrical, thermal, optical, and chemical properties [6]. Consequently, these types of materials have found application in various industrial processes and consumer products, such as drug carriers [2,7], biosensors [8], compounds for bionanocomposites [9,10], energy conversion and storage devices [11,12], environmental purification [13,14], the recovery of valuable compounds such as rare-earth elements and other metals [15,16], etc.

The growing production and application of CNMs has led to a rise in the risk of environmental contamination. CNMs can enter aquatic systems during the life cycle of all products containing CNMs, including during production, transportation, application, and disposal [17]. From this point of view, the risk assessment of CNMs has become a crucial problem for maintaining a safe environment and minimizing human health risks.

Among the main obstacles coupled with risk assessment of nanomaterials (NMs) in the aquatic environment, the following should be highlighted: (1) the dependence of toxic behavior on particle physical and chemical properties, and the variety of sizes, shapes, surface area, functionalization, etc. [18,19,20]; (2) the fate and transformation of NMs in an aquatic environment and in contact with different organisms, which could alter the initial toxicity of materials [21,22]; and (3) species-specific toxicity [23,24]. The following paragraphs briefly discuss these three points.

Particle size, functional groups, oxygen content, surface charges, hydrophobicity, and defect sites can be emphasized among the properties of CNMs defining their toxicity [24]. The generally accepted fact is that shorter CNTs have higher toxicity [25], and SWCNTs are more toxic than MWCNTs [26,27]. Moreover, well-dispersed CNMs are more toxic than their aggregates [28]. The most likely explanation for this size-dependent toxicity is the larger specific surface area of smaller particles and higher interaction with membranes of organisms [29]. The dose–response relationship in the toxicity assessment of CNMs is generally considered not linear, where test organisms may adapt to low concentrations and high concentrations cause a strong negative response [30,31]. The presence of functional groups is a controversial factor that is reported as an inhibitor or stimulator of toxicity compared to pristine unmodified NMs [32,33,34,35]. The surface functionalization of CNMs often changes their main toxic effect between mechanical damage and oxidative stress [32,36].

The environmental transformation of CNMs could significantly change their toxicity. The colloidal behavior and biodegradation of carbon-based and graphene-family nanomaterials in an aquatic environment were reviewed in our previous work [21]. It was previously shown that the addition of natural organic matter (NOM) caused concentration-dependent disaggregation of fullerene C60 crystals and increased potential toxic effects [37]. It also has been reported that the stability of GrO increases in the presence of humic acid and decreases in the presence of polysaccharides [38]. In general, a laboratory toxicity comparison of CNMs is complicated, with variations in the used exposure protocols, including the types of NMs, exposure time, sample preparation methods, etc.

Based on the literature, the sensitivity to CNMs varied between aquatic species, where the most sensitive group of organisms was algae, followed by crustaceans, fish, and bacteria [5]. Considering microalgae as one of the most sensitive organisms to CNMs, it should be mentioned that the toxic effect of CNMs on algal cells might be associated not only with direct exposure but also with shading effect (light absorption and photosynthesis prevention) and nutrient depletion [39]. It is also worth noting that the toxicity of CNMs varies between different microalgae species [40,41]. Several studies have reported the overproduction of extracellular polymeric substances in algae exposed to high concentrations of CNMs as a defensive mechanism [42,43].

This paper focused on the evaluation of toxic levels and toxic effects caused by the impact of four typical representatives of CNMs on marine microalgae. The chosen CNMs were multiwalled carbon nanotubes (CNTs), fullerene (C60), graphene powder (Gr), and graphene oxide (GrO). Microalgae was chosen as a sensitive and useful model for NMs’ environmental risk assessment. In this work, we used a red algae, *Porphyridium purpureum*, which is commonly used in ecotoxicology [44,45,46].

## 2. Materials and Methods

### 2.1. Nanoparticles

In this work, we used four types of CNMs (Table 1), namely multiwalled carbon nanotubes (CNTs), fullerene (C60), graphene powder (Gr), and graphene oxide (GrO). These types of NPs were chosen to compare the toxic effects and the impact of different CNMs on marine microalgae.

### 2.2. Microalgae Cultures and Exposure Protocol

The culture of a red alga, *Porphyridium purpureum* (Bory de Saint-Vincent) Drew et Ross, 1965 (Rhodophyta), originally isolated from Peter the Great Bay (Sea of Japan, Far Eastern Russia) was provided by the Resource Collection “Marine Biobank” of the National Scientific Center of Marine Biology, Far Eastern Branch of the Russian Academy of Sciences (NSCMB FEB RAS). The *P. purpureum* species (Figure S1) was chosen based on their abundance among microalgae in the Sea of Japan [47], and their suitability as test organisms in ecotoxicology [48,49]. The morphology and physiology of *P. purpureum* have been carefully described previously [50,51].

Culturing of microalgae and toxicity test conditions were maintained following the guidance of OECD No.201 [52] with minor modifications, as stated below. Microalgae were cultured with Guillard’s f/2 medium [53]. Filtered (pore diameter of the filter was 0.22 µm) and sterilized seawater with salinity 33 ± 1‰, pH 8.0 ± 0.2 was used for the experiments. The cultivation was carried out at a temperature of 20 ± 2 °C, with an illumination intensity of 300 µmol photons/m^2^/s and a light:dark cycle of 12:12 h.

Before the experiment, microalgae cells were cultivated in 250 mL Erlenmeyer’s flasks. Algal culture in the exponential growth phase was taken for bioassays. For the experiment, microalgae cells were transferred to 12-well plates, where each well contained 2 mL of microalgae aliquots and 2 mL of the tested sample to facilitate. The initial cell density in each well was 5–6 × 10^4^ cells/mL. The wells of the control group had only microalgae aliquots with the addition of 2 mL of f/2 medium. The other wells had different concentrations of the prepared NP suspension.

The stock suspensions of the four used CNMs were prepared in filtered seawater to eliminate additional negative impact on microalgae associated with salinity reduction. The stock concentration for all the CNMs was 250 mg/mL. To prevent the agglomeration of NPs, the stock suspensions were sonicated with ultrasound homogenizer Bandelin Sonopuls GM 3100 (Bandelin Electronic GmbH & Co. KG, Berlin, Germany) with a high-frequency power of 100 W for 30 min. The sonication was performed on ice in 40 mL Sonopuls Rosette cell RZ-2 to prevent sample heating. The prepared stock suspensions were used to obtain the final exposure concentrations of 1, 10, 25, 50, 75, 100, and 125 mg/mL. Each concentration and control group were carried out in triplicate. The duration of microalgae exposure to the NPs was 96 h.

### 2.3. Flow Cytometry Measurement

The method of flow cytometry was used to evaluate the growth rate inhibition, size, and biochemical changes in microalgae cells after exposure to NPs. All the measurements were carried out with CytoFLEX flow cytometer (Beckman Coulter, Indianapolis, IN, USA) with the software package CytExpert v.2.5. Staining by specific fluorescent dyes was used to evaluate biochemical changes and distinguish live and dead microalgae cells. The used endpoints, biomarkers, and parameters of their registration are represented in Table 2. The excitation source and emission channels were selected according to the maximum emission of the used fluorescent dyes, provided by the manufacturer (Molecular Probes, Eugene, OR, USA). In all the cases, the excitation source was a blue laser (488 nm) of the CytoFLEX flow cytometer.

The determination and count of microalgae cells in the analyzed samples were carried out using the parameters of microalgae cell size, granularity, and fluorescence of chlorophyll *a* (emission channel 690 nm). The dead cells were excluded from the count by the presence of intensive fluorescence in the 610 nm emission channel (PI-positive cells).

For the measurement of esterase activity, membrane potential, and ROS generation, after 24 h of exposure, the sample from each well of 12-well plates was gently pipetted, and 500 µL of liquid was transferred to a 48-well plate and stained. The staining was made with PI and one of the other dyes. In general, all the measurements were performed three times separately, namely PI and FDA, PI and FDA, and PI and H_2_DCFDA. PI was used to exclude dead cells, and FDA, DiOC_6_, and H_2_DCFDA were used to assess esterase activity, membrane potential, and ROS generation changes, respectively.

The data of each well was collected at a flow rate of 100 μL/min until 2000 cells were registered. The mean fluorescence intensity (MFI) of the registered cells in the emission channel of 525 nm was used for comparison. For all the types of the used CNMs and each couple of the dyes, negative and positive controls were measured directly before the measurement of the wells with exposed microalgae cells. A negative control group was prepared by 98 °C heat treatment of not-exposed cells in a dry block heater for 15 min. The wells with the addition of only f/2 media were used as a positive control. The obtained MFI data were normalized using positive control as 100% and negative control as 0%.

The growth rate inhibition was measured after 96 h of exposure. The sample from each well of 12-well plates was gently pipetted, and 100 µL of liquid was transferred to a 96-well plate and stained with only PI. The data of each well were collected at a flow rate of 100 μL/min for 30 s. The obtained data were collected as the number of cells per mL and then compared with the number of cells in the control group. The changes in forward scatter intensity and the used size calibration kit allowed us to compare the changes in cell size distribution after exposure to CNMs.

Based on the results of growth rate inhibition, esterase activity, membrane potential, and ROS generation change assays, we calculated the effective concentrations of the CNMs, which caused 10% (EC10) and 50% (EC50) inhibition of listed endpoints. The calculation of EC10 and EC50 values was performed by nonlinear regression fit in GraphPad Prism 8.0.2 (GraphPad Software, San Diego, CA, USA).

### 2.4. Microscopy

Morphological changes of microalgae cells were observed and captured by an optical microscope Axio Observer A1 (Carl Zeiss, Oberkochen, Germany).

### 2.5. Statistical Analysis

Statistical analyses were performed using GraphPad Prism 8.0.2 (GraphPad Software, San Diego, CA, USA). The statistical significance was tested by one-way ANOVA. Normality residuals were checked by the Anderson–Darling test. A value of *p* ≤ 0.05 was considered statistically significant.

## 3. Results

The no-observable-effect level (NOEL) and calculated effective concentrations of the four used CNMs which caused 10% (EC10) and 50% (EC50) inhibition of microalgal growth rate and corresponding changes in esterase activity, membrane potential, and ROS generation in microalgae cells are given in Table 3.

Based on the obtained results, the tested CNMs can be listed depending on the level of toxic exposure in red microalgae *P. purpureum*. Therefore, the growth rate and esterase activity of microalgae reduce in the following order: CNTs > GrO > Gr > C60. Sample CNTs had the highest adverse effect on the growth rate and esterase activity of the microalgae cells. Moreover, only this sample caused membrane depolarization (DiOC_6_ fluorescence decrease) and significantly increased the level of ROS generation (increase in H_2_DCFDA fluorescence) in microalgae cells. Samples C60 and Gr caused high membrane hyperpolarization in microalgae cells (DiOC_6_ fluorescence increase). At the same time, GrO demonstrated no significant effect on cell membrane polarization, even at the highest concentration used. It should be noted that sample C60, which had the lowest toxic impact in the microalgae, was the only type of CNM used that demonstrated no significant effect on ROS generation in the cells of *P. purpureum*. 

**Figure 1:**
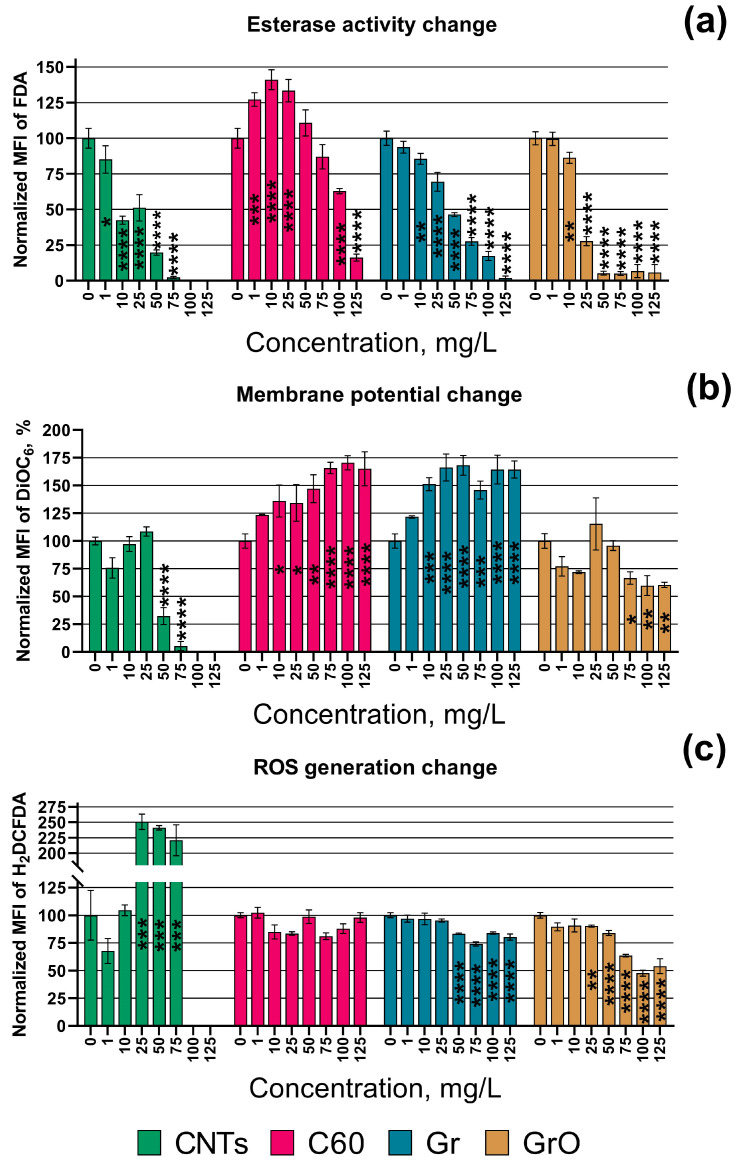
Biochemical changes in microalgae cells after 24 h of exposure to carbon nanomaterials: (**a**) esterase activity; (**b**) membrane potential; (**c**) reactive oxygen species (ROS) generation. MFI, mean fluorescence intensity; FDA, fluorescein diacetate; DiOC6, 3,3′-dihexyloxacarbocyanine iodide; H_2_DCFDA, 2′,7′-dichlorodihydrofluorescein diacetate. *, *p* < 0.05; **, *p* < 0.005; ***, *p* < 0.0005; ****, *p* < 0.0001. The used endpoints were calculated compared to the control, where 0% is negative control (heat-treated cells) and 100% is positive control (cells with no exposure to nanoparticles). The 95% confidence intervals are presented by whiskers.

The changes in esterase activity, membrane potential, and ROS generation depending on the concentration of the CNMs are visualized in Figure 1. The impact of CNTs on esterase activity, membrane potential, and ROS generation in the cells of *P. purpureum* at the concentrations of 100 and 125 mg/L was not represented in Figure 1 because there were no alive microalgae cells at these concentrations. All the data of statistical significance calculations related to these parameters are listed in Table S1.

In addition to the data of Table 3, Figure 1a demonstrated that the esterase activity of *P. purpureum* exposed to C60 increased at lower concentrations (1–25 mg/L), had no significant change compared to the control at middle concentrations (50–75 mg/L), and rapidly decreased at the higher concentrations (100–125 mg/L).

**Figure 2:**
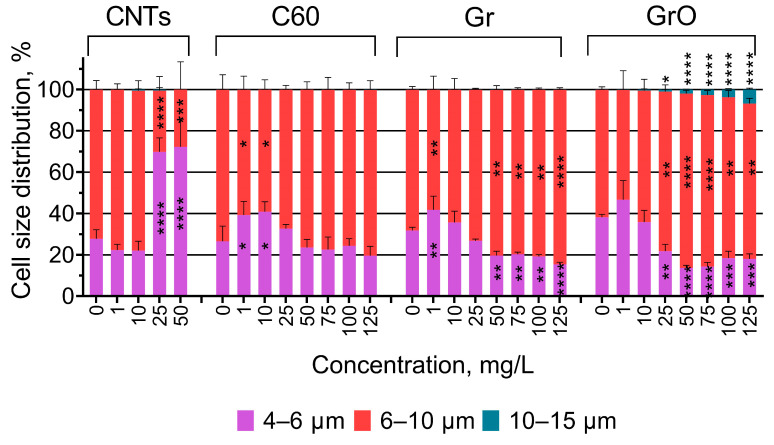
The changes in the size of *P. purpureum* cells after 96 h of exposure to carbon nanomaterials. *, *p* < 0.05; **, *p* < 0.005; ***, *p* < 0.0005; ****, *p* < 0.0001. The 95% confidence intervals are presented by the whiskers.

The changes in the size of *P. purpureum* cells are visualized in Figure 2. The impact of CNTs on the change in cell size of *P. purpureum* at the concentrations of 100 and 125 mg/L was not represented in Figure 2, because there were no live microalgae cells at these concentrations. The data of statistical significance calculations related to the changes in cell size are listed in Table S2.

The highest toxic effect caused by the exposure of *P. purpureum* to CNTs (Table 3, Figure 1) correlates with a decrease in the size of microalgae cells at concentrations of 25 and 50 mg/L. The low concentrations of samples C60 (1–10 mg/L) and Gr (1 mg/L) also caused a moderate decrease in cell size. The higher concentrations of samples Gr (50–125 mg/L) and GrO (25–125 mg/L) caused a noticeable increase in cell size. The most pronounced increase in cell size was observed after the exposure to GrO, and the concentrations of 25–125 mg/L evoked enlargement of the cells to a size of more than 10 µm.

**Figure 3:**
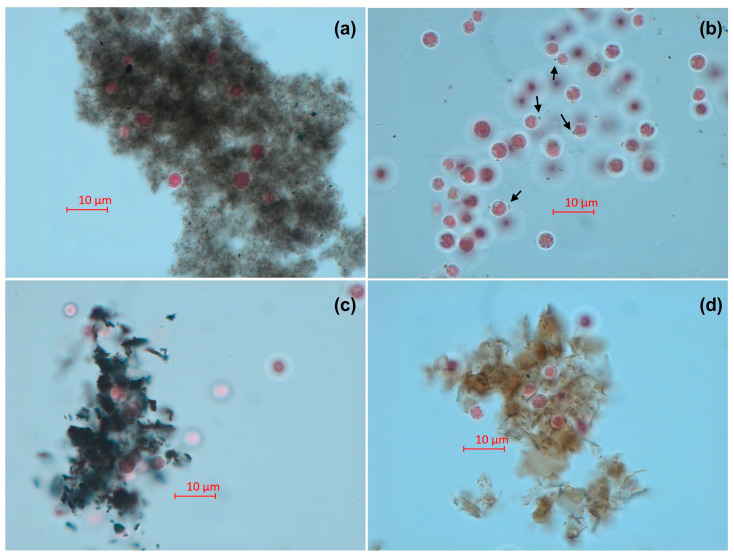
Microscopic pictures of *P. purpureum* after 96 h of exposure to carbon nanomaterials: (**a**) multiwalled carbon nanotubes (sample CNTs) at a concentration of 50 mg/L; (**b**) fullerene (sample C60) at a concentration of 125 mg/L; (**c**) graphene powder (sample Gr) at a concentration of 125 mg/L; (**d**) graphene oxide (sample GrO) at a concentration of 125 mg/L. Black arrows indicate the agglomeration of C60 with the cells. Magnification: 1000×.

Microscopic pictures of *P. purpureum* after 96 h of exposure to CNMs are demonstrated in Figure 3. For all the used CNMs, we chose the highest concentrations with live microalgae cells, namely 50 mg/L for CNTs and 125 mg/L for all the other samples.

The microscopic observation demonstrated that the cells of *P. purpureum* can agglomerate with the big clusters of CNMs. This effect occurred in the cases of CNTs (Figure 3a), Gr (Figure 3c), and GrO (Figure 3d). C60 did not form clusters and demonstrated lower affinity to *P. purpureum* cells, but small particles of C60 were absorbed to the surface of microalgae cells (black arrows in Figure 3b).

## 4. Discussion

Although CNMs are assumed as substances with relatively low toxicity [17,54], they have a great variety of allotropic forms, which could demonstrate different toxic properties in different species and conditions [55,56]. The present study was designed to determine the differences in the effect of multiwalled carbon nanotubes, fullerene, graphene powder, and graphene oxide in marine microalgae *P. purpureum*.

In our previous work, red algae *P. purpureum* was more sensitive to the exposure of CNTs compared to the other marine microalgae species, because of the highly hydrophobic surface of *P. purpureum* cells covered with exopolysaccharide coverage [40]. It is known that the surface properties of CNMs are one of the determinant factors of their toxicity [57,58]. The properties of graphene-family nanomaterials and related biological interactions were carefully described in the work of Sanchez et al. 2011 [59]. Fu and Zhang (2018) in their work explained the relationship between the adhesion and hydrophobicity of NPs [60]. Consequently, hydrophobic NPs would have a higher affinity to hydrophobic regions of the cell membrane, and result in higher potential for accumulation and penetration across the cells [60,61]. 

In this work, none of the used CNMs had any functionalization or surface coatings, and they initially had hydrophobic properties. Among the tested samples, the highest hydrophobicity demonstrated sample CNTs, which rapidly agglomerated in seawater after sonication at high concentrations, and probably had higher adhesion with *P. purpureum* cells. This assumption correlates with the fact that CNTs were found to cause significantly higher toxic effects (Table 3) in the microalgae compared to the other used nanomaterials. Moreover, fullerene C60 revealed the lowest toxicity toward microalgae cells (Table 3) and was the only used CNM that did not form clusters in seawater, even at the highest concentration used (125 mg/L), and demonstrated lower adhesion with the cells (Figure 3b).

It is known that graphene-family NMs could directly penetrate the cell membrane of algae through cell pores [62,63,64]. It was reported that GrO enters into the cells of *Chlorella vulgaris* and damage organelles, enhanced the generation of ROS, and disrupted antioxidant enzymes [65]. On the contrary, our study revealed a decrease in ROS generation in *P. purpureum* under exposure to GrO (Figure 1c). 

As demonstrated in Figure 3, the agglomerated flakes of CNTs, Gr, and GrO were sedimented, and covered microalgae cells. In this case, it is important to notice the role of the shading effect in the toxicity of CNMs. In photosynthetic microorganisms such as microalgae or cyanobacteria, CNMs prevent the illumination of the cells due to their light absorption [41,66]. This effect could limit the photosynthetic activity and growth rate of the microalgae cells and cause metabolic disruption [41]. This effect might be the reason for the observed cell size change in *P. purpureum* exposed to high concentrations of CNTs, Gr, and GrO (Figure 2).

It is known that unlike graphene-family nanomaterials, CNTs are grown catalytically, and often contain residual metal catalysts [59]. The presence of trace metal residuals is another factor that causes higher toxicity of CNTs in microalgae [67]. The used sample of CNTs contained residuals of Al and Co (Table 1). Although Co is one of the essential metals for cell function, it may become toxic at high concentrations [68,69]. It was reported that Al induced oxidative stress, ultrastructural changes, changes in lipid metabolism, degradation of cellular organelles, and suppression of antioxidant enzymatic activity in microalgae [70,71]. These facts can explain the ROS generation increase in *P. purpureum* cells after exposure to CNTs (Figure 1c), as well as the further destruction in proteins, lipids, and carbohydrates, which lead to oxidative stress in microalgae [69].

Despite the assumption of the role of metal catalysts residuals on the toxicity of CNTs, the bioavailability and impact of trace metal inclusions on microalgae ROS generation and toxicity is ambiguous. It was shown that even purified CNTs with almost no metal impurities can cause inflammation and oxidative stress in mice and [72,73]. However, it should be noted that the most of the studies with the microalgae model did not directly evaluate the effect and bioavailability of metal impurities in CNMs on the general toxicity of the tested materials [40,74]. Yin et al. (2020) evaluated the different metal-modified nanocomposites of reduced graphene oxide in the microalgae *Scenedesmus obliquus* and *Chlamydomonas reinhardtii* and demonstrated that a more hydrophobic algal cell surface led to more metal ion adsorption and interactions with NPs [75], which correlates with the highest observed sensitivity of *P. purpureum* (Table 3) to CNTs having the highest concentrations of trace metal residuals among the tested samples (Table 1).

Munk et al. (2017), in their work with green microalgae *Klebsormidium flaccidum*, concluded that ROS production is one of the most important factors that contribute to the harmful effects of MWCNTs on microalgae [76]. At the same time, the authors claimed that MWCNTs had no shading effect on the filamentous microalgae *K. flaccidum*, and did not alter the photosynthetic efficiency of microalgae cells despite the observed aggregation of MWCNTs with the cells [76]. The other work demonstrated that oxidized MWCNTs and GrO caused esterase enzyme inhibition, but no oxidative stress, in cyanobacteria *Microcystis aeruginosa*, and EC50 level 7.38 and 11.1 mg/L for CNTs and GrO, respectively [77]. These results are in agreement with the results of the current study (Table 3) in that the CNTs had higher cytotoxicity in the microalgae than GrO. Several works have shown that Gr is more toxic than CNTs in the microalgae *S. obliquus* and *Chlorella pyrenoidosa* [39,78]. This difference might be associated with particle properties, because Das et al. (2023) used functionalized MWCNTs with a diameter of around 34 nm [78], which is bigger than the unfunctionalized CNTs used in this study (Table 1). The study of Zhang et al. (2018) with *S. obliquus* stated a higher toxicity of Gr compared to GrO [62]. The results of these studies did not agree with our work as the sensitivity of *P. purpureum* to the tested CNMs had the following order: CNTs > GrO > Gr > C60. In the case of Zhang et al. (2018), the sizes of both used CNMs were equal (thickness: 0.8–1.2 nm; diameter: 0.5–2.0 µm) and smaller than Gr and GrO samples used in the current work (Table 1). These differences do not allow us to determine either the higher toxicity of Gr associated with particle sizes or the different sensitivity of *P. purpureum* and *S. obliquus*. Current observations reveal the importance of a multispecies toxicity assessment and the need for the assessment of multiple CNMs with either the same sizes and different surface modifications, or vice versa.

Considering the surface properties of CNMs in aquatic environments, it should be highlighted that NPs inevitably undergo not only physical transformation (agglomeration, sedimentation, etc.) but also surface modification, as a result of interaction with NOM, the absorption of proteins, and “biomolecular corona” formation [21,79]. This issue has attracted the attention of the scientific community [80,81] and requires further study to extend the understanding of the fate and toxicity of CNMs. The “biomolecular corona” formation was not assessed in current work; nevertheless, this phenomenon should be taken into account in further study.

## 5. Conclusions

This study revealed different levels and toxic effects of four CNMs, namely multiwalled carbon nanotubes, fullerene, graphene powder, and graphene oxide, in the microalgae *P. purpureum.* In general, the growth rate and esterase activity of the microalgae reduced in the following order: CNTs > GrO > Gr > C60. All the used CNMs, except fullerene C60, strongly agglomerated in seawater, forming relatively big clusters and agglomerating with microalgae cells, facilitating mechanical damage and metabolic disorder, which was most likely associated with the shading effect. CNTs were the only samples that caused an increase in ROS generation by microalgae cells, which apparently was associated with the higher affinity between the tested NPs and *P. purpureum* cells. The finding of this study highlight the importance of the surface properties of CNMs and microalgae cells in toxicity bioassays. The following studies should consider the interaction of CNMs with NOM and the problem of “biomolecular corona” formation.

## Figures and Tables

**Table 1:** Characteristics of the used carbon nanomaterials.

Sample	Size	Purity	Synthesis or Manufacturer Information
CNTs	Diameter: 6–13 nm; Length: 2.5–20 µm	>98% (Trace metals—13,567 mg/kg, including Al—10,000 mg/kg, Co—2652 mg/kg)	Product Number: 698849; Lot Number: MKCM1457; Sigma Aldrich, St. Louis, MO, USA
C60	Diameter: 0.8 nm	>95.5% (oxide C60)	Batch Number: 120722; Modern Synthesis Technology (MST), Saint-Petersburg, Russia
Gr	Thickness: 3–10 nm; Diameter: 0.5–10 µm	>99%	Type #1, CAS#: 1034343-98-0; Modern Synthesis Technology (MST), Saint-Petersburg, Russia
GrO	Diameter: 10–100 µm	Carbon: 46%; Oxygen: 49%; Hydrogen: 2.5%; Sulfur: 2.5%	CAS#: 1034343-98-0; Modern Synthesis Technology (MST), Saint-Petersburg, Russia

**Table 2:** Bioassay endpoints and registration parameters.

Endpoint	Fluorescent Dye or Registered Parameter	Duration of Microalgae Exposure before the Measurement	Dye Concentration/Duration of Staining *	Emission Channel/Band Width, nm
Growth inhibition	PI	96 h	20 µM/20 min	610/20
Size	Forward scatter intensity (size calibration kit F13838 by Molecular Probes, Eugene, OR, USA)	96 h	–	FSC
Esterase activity	FDA	24 h	100 µM/20 min	525/40
Membrane potential	DiOC_6_	24 h	5 µM/20 min	525/40
ROS generation	H_2_DCFDA	24 h	100 µM/40 min	525/40

ROS, reactive oxygen species; PI, propidium iodide; FDA, fluorescein diacetate; DiOC6, 3,3′-dihexyloxacarbocyanine iodide; H_2_DCFDA, 2′,7′-dichlorodihydrofluorescein diacetate. * The optimization of the used concentrations and duration of staining was described in our previous work [49].

**Table 3:** The toxicity descriptors of CNM exposure to *P. purpureum*, mg/L.

Descriptor	CNTs	C60	Gr	GrO
***Growth rate inhibition*** **, 96 h**				
NOEL, mg/L	<1	50	10	<1
EC10, mg/L	0.49 (0.44–0.55)	24.10 (10.24–67.41)	15.55 (9.48–22.97)	8.60 (7.73–9.55)
EC50, mg/L	2.08 (1.94–2.25)	>131.0	94.88 (83.68–108.50)	23.37 (21.84–24.98)
***Esterase activity inhibition*** **, 24 h**				
NOEL, mg/L	<1	<1	1	1
EC10, mg/L	1.01 (0.36–2.43)	57.12 (41.60–73.18)	14.48 (10.09–19.88)	8.44 (6.84–10.45)
EC50, mg/L	8.18 (5.01–12.44)	93.17 (83.68–102.60)	44.73 (38.82–50.92)	18.28 (16.67–20.02)
***Membrane potential change*** **, 24 h**				
NOEL, mg/L	25 ^inh^	1 ^sti^	<1 ^sti^	125 ^n/a^
EC10, mg/L	38.68 (26.24–50.00) ^inh^	<1 ^sti^	<1 ^sti^	n/a
EC50, mg/L	46.55 (39.39–49.55) ^inh^	17.40 (6.76–32.61) ^sti^	5.61 (1.16–12.69) ^sti^	n/a
***ROS generation change*** **, 24 h**				
NOEL, mg/L	10 ^sti^	125 ^n/a^	25 ^inh^	25 ^inh^
EC10, mg/L	10.82 (n/a)	n/a	27.90 (13.80–43.82) ^inh^	25.54 (13.64–37.77) ^inh^
EC50, mg/L	14.66 (n/a)	n/a	>125 ^inh^	123.20 (106.30–153.00) ^inh^

^inh^, inhibition effect; ^sti^, stimulative effect; ^n/a^ or n/a, not assessed (the descriptor cannot be calculated). EC10 and EC50 are represented as mean values, with 95% confidence intervals presented in the parenthesis.

## Data Availability

Not applicable.

## Supplementary Materials

The following supporting information can be downloaded at: https://www.mdpi.com/article/10.3390/toxics11060491/s1, Figure S1: Microscopic picture of *P. purpureum* from the control group; Table S1: The statistical significance calculation of esterase activity, membrane potential, and ROS generation in *P. purpureum* cells after 24 h of exposure; Table S2: The statistical significance calculation of the changes in *P. purpureum* cells after 96 h of exposure.
