# Supplementary material for: The Comparative Toxic Impact Assessment of Carbon Nanotubes, Fullerene, Graphene, and Graphene Oxide on Marine Microalgae Porphyridium purpureum
Source: Toxics. 2023 May 30;11(6):491. doi: 10.3390/toxics11060491 (PMC10304638; doi:10.3390/toxics11060491)
Supplement: Supplementary file 1 [file toxics-11-00491-s001.zip › toxics-2409768-supplementary.pdf]

The Comparative Toxic Impact Assessment of Carbon Nanotubes, Fullerene, Graphene, and Graphene Oxide on Marine Microalgae *Porphyridium purpureum*

Konstantin Pikula [1,*], Seyed Ali Johari [2], Ralph Santos-Oliveira [3,4] and Kirill Golokhvast [1,5]

[1] Polytechnical Institute, Far Eastern Federal University, 10 Ajax Bay, Russky Island, Vladivostok 690922, Russia; golokhvast@sfsca.ru
[2] Department of Fisheries, Faculty of Natural Resources, University of Kurdistan, Pasdaran St, Sanandaj 66177-15175, Iran; sajohari@gmail.com
[3] Laboratory of Nanoradiopharmaceuticals and Synthesis of Novel Radiopharmaceuticals, Nuclear
Engineering Institute, Brazilian Nuclear Energy Commission, Rua Hélio de Almeida 75, Rio de Janeiro 21941906, Brazil; roliveira@ien.gov.br
[4] Laboratory of Nanoradiopharmaceuticals and Radiopharmacy, Rio de Janeiro State University,
R. São Francisco Xavier, 524, Rio de Janeiro 23070200, Brazil
[5] Siberian Federal Scientific Center of Agrobiotechnology RAS, Centralnaya Str., Presidium, Krasnoobsk 633501, Russia
* Correspondence: k.pikula@mail.ru

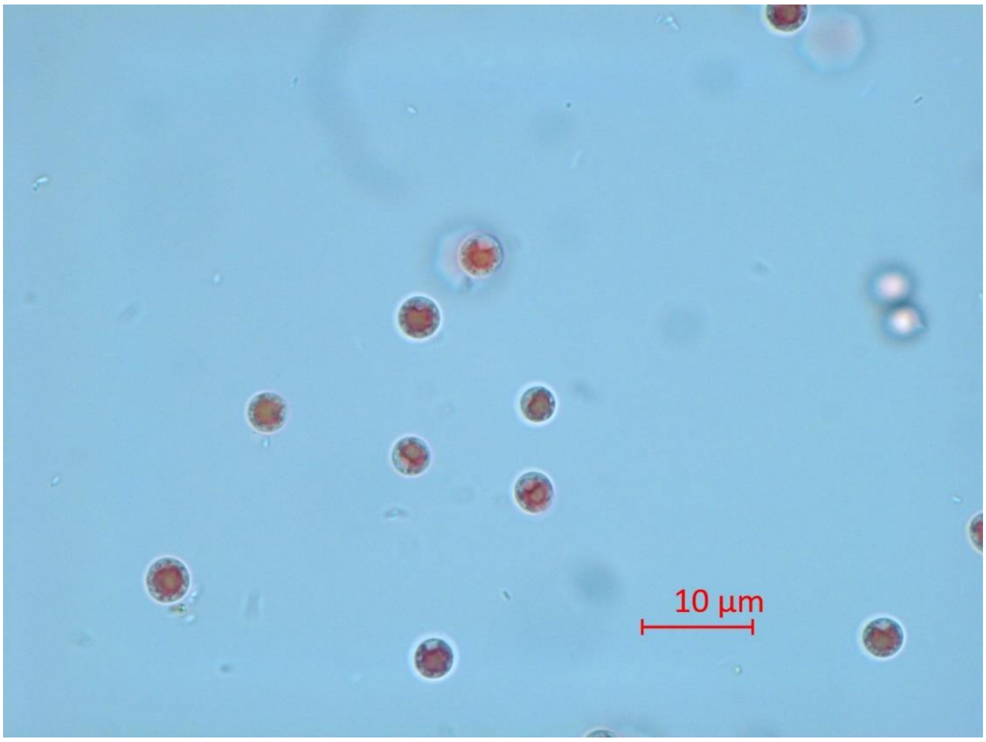

Figure S1. Microscopic picture of *P. purpureum* from the control group.

Table S1. The statistical significance calculation of growth rate, esterase activity, membrane potential, and ROS generation changes in *P. purpureum* cells.

Concentration. mg/L	CNTs		C60		Gr		GrO	
**Growth rate inhibition, 96 h**								
1	***	0.0004	ns	0.6562	ns	0.4119	****	<0.0001
10	****	<0.0001	ns	0.7143	ns	0.0883	****	<0.0001
25	****	<0.0001	ns	0.6845	**	0.0049	****	<0.0001
50	****	<0.0001	ns	0.2042	****	<0.0001	****	<0.0001
75	****	<0.0001	*	0.0454	****	<0.0001	****	<0.0001
100	n/a	<0.0001	**	0.0057	****	<0.0001	****	<0.0001
125	n/a	<0.0001	*	0.0118	****	<0.0001	****	<0.0001
**Esterase activity change, 24 h**								
1	*	0.0224	***	0.0007	ns	0.2676	ns	0.9998
10	****	<0.0001	****	<0.0001	**	0.0018	**	0.0032
25	****	<0.0001	****	<0.0001	****	<0.0001	****	<0.0001
50	****	<0.0001	ns	0.2647	****	<0.0001	****	<0.0001
75	****	<0.0001	ns	0.1285	****	<0.0001	****	<0.0001
100	n/a	<0.0001	****	<0.0001	****	<0.0001	****	<0.0001
125	n/a	<0.0001	****	<0.0001	****	<0.0001	****	<0.0001
**Membrane potential change, 24 h**								
1	ns	0.2576	ns	0.1437	ns	0.1169	ns	0.1001
10	ns	0.9020	*	0.0148	***	0.0004	ns	0.0617
25	ns	0.1008	*	0.0207	****	<0.0001	ns	0.3921
50	****	<0.0001	**	0.0020	****	<0.0001	ns	0.9951
75	****	<0.0001	****	<0.0001	***	0.0009	*	0.0106
100	n/a	<0.0001	****	<0.0001	****	<0.0001	**	0.0024
125	n/a	<0.0001	****	<0.0001	****	<0.0001	**	0.0067
**ROS generation change, 24 h**								
1	ns	0.6425	ns	0.4558	ns	0.6279	ns	0.0756
10	ns	0.9997	ns	0.4631	ns	0.5122	ns	0.1290
25	***	0.0002	ns	0.3701	ns	0.2023	ns	0.1007
50	***	0.0004	ns	0.9998	****	<0.0001	**	0.0029
75	***	0.0003	ns	0.2486	****	<0.0001	****	<0.0001
100	n/a	0.0109	ns	0.6707	****	<0.0001	****	<0.0001
125	n/a	0.0337	ns	0.9997	****	<0.0001	****	<0.0001

ROS, Reactive oxygen species; *, $p < 0.05$; **, $p < 0.005$; ***, $p < 0.0005$; ****, $p < 0.0001$; n/a, not assessed; ns, nonsignificant ($p > 0.05$).

Table S2. The statistical significance calculation of the changes in the size of *P. purpureum* cells after 96 h of exposure to carbon nanomaterials.

Concentration, mg/L	4-6 μm		6-10 μm		10-15 μm	
**CNTs**						
1	ns	0.7792	ns	0.7875	ns	0.9993
10	ns	0.7496	ns	0.8065	ns	0.3760
25	***	0.0002	***	0.0001	ns	0.5322
50	****	<0.0001	***	0.0001	ns	0.9988
75	n/a	n/a	n/a	n/a	n/a	n/a
100	n/a	n/a	n/a	n/a	n/a	n/a
125	n/a	n/a	n/a	n/a	n/a	n/a

**C60**						
1	*	0.0404	*	0.0390	ns	0.9978
10	*	0.0184	*	0.0178	ns	0.8838
25	ns	0.5608	ns	0.5615	ns	0.8495
50	ns	0.9631	ns	0.9589	ns	0.9137
75	ns	0.8700	ns	0.8708	ns	0.9589
100	ns	0.9924	ns	0.9919	ns	0.9997
125	ns	0.4260	ns	0.4262	ns	0.8115
**Gr**						
1	**	0.0090	**	0.0088	ns	0.9999
10	ns	0.5525	ns	0.5537	ns	0.9936
25	ns	0.3239	ns	0.3188	ns	0.9977
50	**	0.0018	**	0.0018	ns	0.9977
75	**	0.0029	**	0.0028	ns	0.9317
100	**	0.0013	**	0.0013	ns	0.9999
125	****	<0.0001	****	<0.0001	ns	0.7740
**GrO**						
1	ns	0.1397	ns	0.1320	ns	0.9999
10	ns	0.9784	ns	0.9911	ns	0.5981
25	**	0.0019	**	0.0028	*	0.0183
50	****	<0.0001	****	<0.0001	****	<0.0001
75	****	<0.0001	***	0.0001	****	<0.0001
100	***	0.0003	**	0.0019	****	<0.0001
125	***	0.0003	**	0.0089	****	<0.0001

ROS, Reactive oxygen species; *, $p < 0.05$; **, $p < 0.005$; ***, $p < 0.0005$; ****, $p < 0.0001$; n/a, not assessed; ns, nonsignificant ($p > 0.05$).
